# Reporting and recording of adverse drug reactions of highly active antiretroviral therapy by HIV infected patients and healthcare professionals respectively in the Ethekwini Metropolitan of Kwa-Zulu Natal, South Africa: a cross-sectional and retrospective comparative study

**DOI:** 10.11604/pamj.2022.42.218.32239

**Published:** 2022-07-20

**Authors:** Sindiswa Landile Zondi, Panjasaram Naidoo

**Affiliations:** 1 Discipline of Pharmaceutical Sciences, School of Health Sciences, University of Kwa-Zulu Natal, Durban, South Africa

**Keywords:** HIV, adverse drug reactions, HAART, reporting, adverse drug effects, health care professionals

## Abstract

**Introduction:**

even though Highly Active Antiretroviral Therapy (HAART) is effective in managing Human Immuno-deficiency Virus (HIV) infection, it is not without its adverse drug effects (ADE) and or adverse drug reactions (ADRs). The study of ADRs associated with HAART in hospitals and clinics is crucial in gauging the burden of the severity of morbidity and mortality in such facilities, hence the reporting of such ADRs is important.

**Methods:**

the study was divided into 2 phases: the 1^st^ phase entailed collecting data from HIV infected patients using a questionnaire on ADR experienced, whilst the 2^nd^ phase was a retrospective analysis of respective patients´ medical files to record if an ADR was experienced. Three antiretroviral clinics linked to public sector facilities in EThekwini Metro, Kwa-Zulu Natal were the study sites.

**Results:**

seventy-two percent of patients reported at least one ADR after HAART initiation. Skin rash (11%) was the most commonly stated ADR by patients, whilst anemia (29%) and cardiovascular disease (23%) were the most commonly recorded ADRs on the patients´ medical files. Of those patients who reported ADRs, 57% were on the first line regimen consisting of Tenofovir, Emtricitabine and Efavirenz. Thirty-six patients reported that they were admitted to hospitals due to ADRs, however none resulted in death. These ADRs were experienced by patients on different regimens, with 10 admissions from the same regimen.

**Conclusion:**

adverse drug reactions were experienced by South African patients, however the reporting of ADRs by patients were inconsistent with what was recorded on their medical files.

## Introduction

South Africa has one of the highest HIV and AIDS prevalence in the world, according to the Joint United Nations Programme on HIV and AIDS (UNAIDS) report; the Eastern and Southern African region accounts for the highest number of people living with HIV, i.e 20.7 million [1]. Furthermore, there are 7.5 million people living with HIV and AIDS in South Africa [2]. Access to HAART has improved the quality of life and chances of survival in people living with HIV [3]. Sustained suppression of viral replication by HAART has led to a decrease in disease progression, resulting in positive clinical outcome for HIV infected patients [4]. However, coupled with these clinical benefits are unwanted effects known as ADRs. Medicines can be summarized according to its risk, benefit and quality [5]. One of the risks of medicine are ADRs. An ADR is defined as 'an appreciably harmful or unpleasant reaction, resulting from an intervention related to the use of a medicinal product, which predicts hazard from future administration and warrants prevention or specific treatment, or alteration of the dosage regimen, or withdrawal of the product' [6]. Another definition of an ADR is a “response to a drug which is noxious and unintended and which occurs at doses normally used in man for prophylaxis, diagnosis, or therapy of disease or for the modification of physiologic function” [7]. A causal link exists between a drug and an ADR [7]. In summary, it can be said that an ADR is harm directly caused by the drug, in this case HAART at normal doses, during normal use [7].

Adverse drug reactions are classified into six types (with mnemonics): dose-related (augmented), non-dose-related (bizarre), dose-related and time-related (chronic), time-related (delayed), withdrawal (end of use), and failure of therapy (failure) [6]. Timing, the pattern of illness, the results of investigations, and re-challenge can help attribute causality to a suspected ADRs [7]. Whilst an antibody-dependent enhancement (ADE) is “an injury resulting from the use of a drug”. Under this definition, the term ADE includes harm caused by the drug (ADRs and overdoses) and harm from the use of the drug (including dose reductions and discontinuations of drug therapy) [8]. Adverse drug reactions can be caused by any therapeutic agent, including prescribed and over the counter (OTC) medicines, vaccines, and complementary medicines, and all of these should be reported [9]. Adverse drug reactions are a major public health concern as they cause morbidity, mortality and high financial burden [10]. Adverse drug reactions are prevalent in South Africa such that 1 in 12 hospital admissions are due to ADRs, which also accounts for the 16% of death in adult medical admissions, hence the ongoing search and the need to transform clinical practice [5]. Adverse drug reactions in developing countries can be vast compared to those in developed countries due to other conditions such as Tuberculosis (TB), poverty, malnutrition and genetic predispositions [11]. There are other factors that contribute to the development of ADRs e.g age, gender, treatment duration, cluster of differentiation 4 (CD4) count, viral load and body mass index [12]. Studies have established a correlation between age, gender and response to treatment i.e. women are more likely to develop hepatotoxicity and rash, which is a risk factor, whereas patients that are above the age of 40 are prone to developing neuropathy and other adverse reactions and that females are more likely to encounter ADRs compared to males [4,13].

In another study on children by Mouton it was found that of 1050 patients (median age 11 months, 56% male, 2.8% HIV-infected) with 1106 admissions; 40 were serious ADRs (3.8 per 100 drug-exposed admissions), including 9/40 (23%) preventable serious ADRs, and 8/40 (20%) fatal or near-fatal serious ADRs [14]. Antibacterials, corticosteroids, psycholeptics, immuno-suppressants, and antivirals were the most commonly implicated drug classes [14]. Preterm neonates and children in middle childhood (6 to 11 years) were at increased risk of serious ADRs compared to infants (under 1 year) and term neonates [14]. The HIV clinical stages as stated by the World Health Organization (WHO) also play a role in the development of ADRs [15]. Patients in the advanced stages are more prone to ADRs than patients in clinical stage 1 [15]. This is due to the fact that most patients in advanced stages have co-morbidities, leading to drug-drug interactions with overlapping toxicity and pill burden, which leads to inefficient treatment [15-17]. Adverse drug reactions have limited the success of HAART as they are responsible for the co-morbidities seen in such patients, resulting in decreased adherence to treatment and consequent virological failure and poor prognosis [18]. Human Immuno-deficiency Virus (HIV) being a chronic disease means the patient has to have continuous exposure to HAART and the resultant ADRs. This has resulted in the patient/ caregiver having limited options such as either decreasing the dosage of antiretroviral drugs thus compromising efficacy, withdrawing the offending drug and substituting it with another drug, or symptomatically treating the ADRs [4]. Substituting the offending drug becomes problematic, especially in resource limiting settings as the regimens exist in fixed dose combination [4]. Other studies have shown that ADRs are present during the early stages of ARV initiation and these ADRs led to reduction in the adherence to HAART [12,19,20]. Visible adverse drug reactions such as buffalo hump, excessive sweating, darkening of the skin, hair loss, skin rash, body odor, led to low self-esteem which eventually led to poor adherence to HAART [19]. A review by Li *et al*. 2017 suggested that pre-counselling of HIV patients and the pre-knowledge of the expected ADRs and how these reactions should be managed led to less non-adherence [19]. It was then suggested that HIV patients on HAART should undergo pre-counselling and be informed of potential ADRs [15,21].

A study conducted by Manickum and Suleman, 2012, revealed that there were 3534 patients in Kwa-Zulu Natal between May 2007 and May 2008 that experienced ADRs [22]. According to the South African Health Products Authority (SAHPRA) nearly 5% of hospitalized patients experience an ADE, making them one of the most common types of inpatient errors [9]. The ten most common ADRs experienced were constipation, nausea/vomiting, fatigue, alopecia, drowsiness, myelosuppression, skin reactions, anorexia, mucositis and diarrhoea [9]. These ADRs have high-documented incidence rates and were also the ten most predictable ADRs in this study [9]. The putative effects of HAART in HIV patients leads to the development of ADRs/ADEs which have been associated with the decrease in HAART adherence, hence it is important to strengthen patient´s drug safety, improve adherence and treatment outcomes through the proper management of ADRs/ADEs. It is imperative to deduce for each patient whether the responsible drug elicited an adverse reaction or event, or an expected effect. It is also imperative to infer if the adverse drug reactions or events in the patients are due to recreational drug abuse or drug to drug interactions, as there is an ongoing concern of ARV and other drugs misuse and drug to drug interactions effects. The study of medical drugs to drugs (HAART) interactions is important, as the results may lead to suboptimal activity of HAART or toxicity. The objective of this study therefore is to investigate the number of ADRs experienced by patients on HAART.

## Methods

**Study design:** the study was divided into 2 phases. The first phase was a quantitative cross-sectional study, whilst the second phase was a retrospective analysis of patient´s medical files. The questionnaire was developed by doing an extensive literature search (published and unpublished) and accessing questionnaires from these studies. A similar format was used, however the questions or variables were designed to meet the objectives set out in the protocol and relevant to the diverse population of South Africa. For each objective, confirmation was sought that a question was formulated to obtain the relevant data. Regarding the pilot study, questionnaires were pre-tested by administering them to post graduate students of different races, studying at the university. This was to ensure that the questions were not ambiguous, they were easy to understand, and that the time taken to complete the questionnaire was reasonable. The questionnaires were then amended according to the majority input by the postgraduate students. The questionnaire then received final approval from the supervisor. Psychometric testing of the questionnaires were however not carried out, which will be described as a limitation.

**Study setting:** the study was undertaken at 3 public sector hospitals that had an antiretroviral (ARV) outpatient clinic attached to the hospital. These hospitals were situated in the eThekwini Metropolitan District of Kwa-Zulu Natal, South Africa. The total target population of patients using HAART in the eThekwini Metropolitan (Metro) was, 383869 during the time of the study [23]. The cross-sectional study was conducted from December 2019 to January 2020, whilst the retrospective study analysis dated back from 1991-2019.

**Study population and determination and selection of sample size:** all patients attending the study sites formed the study population. Sample size was calculated using single population proportion formula,


$$n=\frac{\frac{Z^{2}P(1-P)}{d^{2}}}{1+\frac{Z^{2}P(1-P)}{Nd^{2}}}$$

using 95% confidence level, 5% degree of precision, 50% of expected number of patients with number of adverse reactions and the total target population of patients using HAART in the Metro (383869) [23,24]. The calculated sample size was 384. Taking into consideration the possibility of drop-outs and unforeseen circumstances, 10 percent was added to the sample to make a maximum sample size of 423. The maximum overall sample size was distributed equally amongst the three public hospitals, i.e the sample size for each hospital was 141. Prior to commencing the study, permission was sought from the hospitals and ethical approval was obtained from the University of Kwa-Zulu Natal Ethics Committee with ethics approval number BE053/19.

**Sample recruitment:** during the study period, whilst patients' were awaiting consultation with the doctor or nurse, these patients were approached as a group and briefed on the study with the intention of getting their participation in the study. In addition, informed consent, voluntary participation, anonymity and any queries were also addressed. The inclusion criteria was communicated to them (all patients that were on HAART for 6 months or longer were eligible for the study). Those who consented to participate were given a coded, closed ended, anonymous questionnaire to fill. The purpose of the coding was to match the questionnaire to the patient's clinical file, which was the second phase of the study. The code was immediately written on the patient´s medical file. The questionnaire was available in both English and IsiZulu the latter being the most common language spoken in the eThekwini Metro and South Africa [25]. The questionnaire required information on their demographics, their drug history and confirmation of ADRs experienced. All the data collected was treated confidentially.

**Data analysis:** the data was analysed using SPSS version 25. Continuous and categorical variables were analysed. Categorical variables such as race and gender were compared using descriptive analysis as appropriate. The level of significance was at p < 0.05.

## Results

**Response rate and demographics:** the response rate was 100%. Four hundred and twenty-six (426) completed questionnaires were received and analysed, of which 296 (69%) respondents were females, 126 (30%) were males and 4 (1%) were transgender respondents. The ages of the respondents ranged from 18 to 69, with the median age of 41 years (IQR 39.5 to 41.7).

**Regimens that led to ADRs:** Table 1 describes the regimens that led to the different ADRs. Three hundred and six (306) patients (72%) reported at least one ADR with 243 (56%) patients on the first line Tenofovir (TDF), Emtricitabine (FTC) and Efavirenz (EFV) regimen. 36 (9%) patients reported that they were admitted to hospital due to ADRs, but none of the admissions led to death. Ten of the admitted participants were on the TDF+FTC+EFV regimen.

**Table 1:** regimens that led to ADRs (medical files)

Regimen	Number of patients on the regimen n/(%)		Number of patients who from total number that experienced at least one ADR on the regimen n/(%)		Date of regimen initiation	Regimen leading to hospital admission n/(%)
TDF+FTC+EFV	243	57%	174	41%	2012	10 (2%)
TDF+3TC+EFV	48	11%	29	7%	2012	6 (1.4%)
TDF+FTC+LPV/r	5	1%	4	1%	2015	0
TDF+3TC+LPV/r	6	1.4%	6	1.4%	2015	0
TDF+3TC+NVP	15	4%	9	2%	2010	3 (0.7%)
TDF+FTC+NVP	15	4%	12	3%	2015	2 (0.5%)
ABC+3TC+EFV	11	3%	8	2%	2015	4 (0.9%)
ABC+FTC+EFV	4	1%	4	1%	2017	0
ABC+3TC+NVP	1	0.23%	0	0%	2015	0
ABC+3TC+LPV/r	6	1.4%	4	1%	2013	1 (0.23%)
AZT+3TC+LPV/r	58	14%	47	11%	2010	6 (1.4%)
AZT+3TC+ATZ/r	3	0.7%	2	0.5%	2019	1 (0.23%)
AZT+3TC+EFV	9	2%	5	1.2%	2014	3 (0.23%)
AZT+FTC+LPV/r	1	0.23%	1	0.23%	2018	0
AZT+3TC+NVP	1	0.23%	1	0%	2013	0

ABC**:** Abacavir**;** 3TC**:** Lamuvidine**;** EFV**:** Efavirenz**;** D4T: Saquinavir**;** LPV/r: Lopinavir/ritonavir**;** TDF**:** Tenofovir**;** NVP: Nevirapine**;** FTC: Emtricitabine; AZT: Zidovudine; ATZ/r**:** Atazanavir/ritonavir

**Patients self-reported ADRs:** skin rash 47 (11%), headache 40 (9%), nausea 31 (7%), depression 30 (7%) and fever 28 (7%) were the most commonly stated ADRs by patients (Table 2).

**Table 2:** patients' self-reported adverse drug reactions stratified according to age and gender

Gender	Males (n=126)			Females (n=296)			Transgender (n=4)		
Age	18-35years	36-55 years	56+years	18-35years	36-55 years	56+ years	18-35 years	36-55 years	56+ years
Self-reported ADR									
Abdominal pain	-	4 (3%)	-	9 (3%)	10, (3%)	-	-	-	-
Abnormal fat distribution/ lipodystrophy	-	1 (0.8%)	1 (0.8%)	1 (0.3%)	7 (2.4%)	-	-	-	-
Anemia	-	2 (1.6%)	-	4 (1.4%)	5 (1.7%)	-	1 (25%)	-	-
Cardiovascular disease	-	-	-	-	2, (0.7%)	-	-	-	-
Constipation	-	2 (1.6%)	-	6 (2%)	10 (3%)	1 (0.3%)	-	-	-
Depression	4 (3%)	5 (4%)	1 (0.8%)	8 (2.7%)	11 (4%)	1 (0.3%)	-	-	-
Diarrhea	1 (0.8%)	1 (0.8%)	2 (1.6%)	8 (2.7%)	11 (4%)	1 (0.3%)	-	-	-
Enlarged breasts	-	-	-	1 (0.3%)	2 (0.7%)	-	-	-	-
Fever	4 (3%)	3 (2.4%)	-	14 (4.7%)	6 (2%)	1 (0.3%)	-	-	-
Fat gain	1 (0.8%)	-	-	13 (4%)	12 (4%)	1 (0.3%)	-	-	-
Fat loss/lipoatrophy	-	4, (3%)	-	7 (2.4%)	9 (3%)	1, (0.3%)	-	-	-
Headache	5 (4%)	5 (4%)	-	16 (5.4%)	14 (5%)	-	-	-	-
Hearing loss	1 (0.8%)	1 (0.8%)	1 (0.8%)	2 (0.7%)	2 (0.7%)	-	-	-	-
Heart burn	-	1 (0.8%)	-	7 (2.4%)	9 (3%)	2 (0.7%)	-	-	-
Hepatotoxicity	1 (0.8%)	-	1 (0.8%)	-	4 (1.3%)	-	-	-	-
Hyperglycemia	-	-	-	1, (0.3%)	2 (0.7%)	-	-	-	-
Hyperlactatemia	-	1 (0.8%)	-	-	-	-	-	-	-
Loss of appetite	-	1 (0.8%)	, (0.8)	12 (4%)	6 (2%)	-	-	-	-
Nausea or vomiting	-	4 (3%)	-	14 (4.7%)	11 (4%)	2 (0.7%)	-	-	-
Neuropsychiatric effects	1 (0.8%)	-	-	5 (1.7%)	3 (1%)	-	-	-	-
Osteoporosis	-	-	-	4 (1.3%)	-	-	-	-	-
Pancreatitis	-	-	-	-	1 (0.3%)	-	-	-	-
Persistent muscle pain	-	5 (4%)	1 (0.8%)	5 (1.7%)	10 (3%)	6 (2%)	-	-	-
Problem with breathing	-	-	-	3 (1%)	5 (1.7%)	1 (0.3%)	-	-	-
Renal impairment	-	3 (2.4%)	1 (0.8%)	2 (0.7%)	6 (2%)	5 (1.7%)	-	-	-
Skin pigmentation	2 (1.6%)	2 (1.6%)	-	4 (1.3%)	4 (1.3%)		-	-	-
Skin rash	1, (0.8%)	6 (5%)	1 (0.8%)	9 (3%)	23 (8%)	5 (1.7%)	2 (50%)	-	-
Unusual bleeding	-	1 (0.8%)	-	2 (0.7%)	2 (0.7%)	-	-	-	-
Unusual fatigue	-	3 (2.4%)	-	10 (3%)	10 (3%)	1,(0.3%)	-	-	-

**Adverse drug reactions obtained from the healthcare professionals medical files of the respective patients:** anemia 123 (29%), cardiovascular disease 99 (23%), diarrhea 40 (9%), depression 33 (8%) and skin rash 27 (6%) were the most commonly recorded ADRs on the patients´ medical files (Table 3).

**Table 3:** ADRs recorded by HCPs and their subsequent outcomes

Adverse drug reactions	Patients' reported ADRs: n (%)	Adverse drug reactions recorded by HCP: n (%)	P value
Abdominal pain	23 (5.4%)	8 (1.9%)	p= 0.0935
Anemia	11 (2.6%)	123 (29%)	p= 0.0000
Cardiovascular disease	2 (0.5%)	99 (23%)	p= 0.0000
Constipation	19 (4.5%)	6 (0.14%)	p= 0.1560
Depression	28 (6.6%)	33 (7.7%)	p= 0.9997
Diarrhea	24 (6%)	40 (9%)	p= 0.0235
Fever	28 (6.6%)	26 (6.1%)	p= 0.9984
Headache	40 (9.4%)	17 (4%)	p= 0.0038
Nausea or vomiting	32 (7.5%)	23 (5.4%)	p= 0.0029
Neuropsychiatric effects	9 (2%)	19 (4.5%)	p= 0.2097
Osteoporosis	11 (2%)	2 (0.5%)	p=0.6728
Persistent muscle pain	26 (6%)	4 (1%)	p= 0.0000
Renal impairment	15 (3.5%)	2 (0.5%)	p= 0.6161
Skin rash	47 (11%)	27 (6%)	p= 0.0000

**Comparison of ADRs reported by patients versus those recorded by healthcare professionals:** a hundred and twenty-three anemia ADRs (29%) that were recorded in patients´ medical files, compared to 11 (2.6%) that were reported by patients on the questionnaires (p=0.0000) (Table 4). There were 47 (11%) skin rash ADRs that were reported by patients on the questionnaires, compared to 27 that were documented on the patients´ medical files (p=0.0000). Cardiovascular disease ADRs that were reported by patients were lower 2 (0.5%) compared to those that were recorded on the patients´ medical files 99 (23%) (p=0.0000).

**Table 4:** comparison of adverse drug reactions reported by patients versus those recorded by HCPs

Adverse drug reactions	Patients' reported ADRs: n (%)	Adverse drug reactions recorded by HCP: n (%)	P value
Abdominal pain	23 (5.4%)	8 (1.9%)	p= 0.0935
Anemia	11 (2.6%)	123 (29%)	p= 0.0000
Cardiovascular disease	2 (0.5%)	99 (23%)	p= 0.0000
Constipation	19 (4.5%)	6 (0.14%)	p= 0.1560
Depression	28 (6.6%)	33 (7.7%)	p= 0.9997
Diarrhea	24 (6%)	40 (9%)	p= 0.0235
Fever	28 (6.6%)	26 (6.1%)	p= 0.9984
Headache	40 (9.4%)	17 (4%)	p= 0.0038
Nausea or vomiting	32 (7.5%)	23 (5.4%)	p= 0.0029
Neuropsychiatric effects	9 (2%)	19 (4.5%)	p= 0.2097
Osteoporosis	11 (2%)	2 (0.5%)	p=0.6728
Persistent muscle pain	26 (6%)	4 (1%)	p= 0.0000
Renal impairment	15 (3.5%)	2 (0.5%)	p= 0.6161
Skin rash	47 (11%)	27 (6%)	p= 0.0000

## Discussion

A high percentage (72%) of HIV infected patients surveyed during the study period experienced an ADR in the 3 public sector hospitals. These findings correlate with findings by Tadesse *et al*. where 345 (89.9%) of HIV patients reported ADRs [26] and by a study conducted in Brazil, whereby 85.5% of HIV patients reported at least one ADR [27]. The TDF+FTC+EFV regimen initiated in 2012 led to 174 (41%) of patients experiencing an ADR either as reported or recorded in the medical files. As can be seen in Table 1 and Table 2 the females experienced the most number of ADRs. In Mali, Oumar *et al*. found a female predominance in their study as 58% HIV positive female patients reported ADRs [21]. Multiple studies have illustrated sex differences in pharmacokinetics, mainly due to females exhibiting increased body fat, lower body weight and organ size [28]. Thirty six (8.5%) patients were hospitalized due to ADRs and none resulted in death, our findings were correlated by Oscanoa *et al*. who also reported 8.7% ADRs hospital admissions [29].

Skin rash was the most reported (11%) ADR by patients. These findings are similar to findings in a North Indian study, where 22 (10.18%) HIV infected patients reported skin rash [30]. The other commonly experienced ADRs reported by patients were nausea/vomiting and diarrhea. These findings were consistent with findings from an Eritrean study, whereby gastrointestinal disease was the most common ADR amongst HIV patients on antiretroviral treatment [31]. The incidence of neuropsychiatric effects that were reported and recorded by patients and healthcare professionals (HCPs) respectively, is not unusual as the regimen contained efavirenz. Efavirenz is known to be implicated in central nervous system effects, dizziness, neurocognitive impairment and abnormal dreams [32-34]. Data recorded from patient's files included anemia (29%) and cardiovascular disease (23%) as the most common ADR in this study. Amongst the 126 female participants, 57 were diagnosed with unresolved cardiovascular disease, in contrast with 4 male participants. These findings are similar to a meta-analyses study which showed that HIV patients on HAART have a 61% greater relative risk of developing cardiovascular disease than people who have not contracted HIV, furthermore HIV patients on HAART have 2 times greater relative risk of developing cardiovascular disease than HAART naïve HIV patients [35]. Some HAART drugs have been implicated in causing anemia in a prenatal studies based in Thailand [36].

Another commonly recorded effect was nausea/vomiting. Medication induced nausea and vomiting is a commonly known side effect of HAART [37]. The reporting and recording of nausea as an ADR instead of a side effect in studies have demonstrated the possibility of a lack of knowledge as to what an ADR is versus a side effect. This is further confirmed in this study, whereby the number of 'ADRs' reported by patients versus those that were recorded in the medical files differed (29 versus 14). The inverse is also true where patients did not report an effect as ADR, but it was recorded as ADR by the HCP. In the case of anaemia a significant difference was found in what was reported and what was recorded by the HCP (p<0.05). Another example was the cases of recorded cardiovascular disease that were significantly higher than those that were reported by patients (p<0.05). The inconsistency in the ADRs reported by patients and those that are recorded by HCPs in the patient data sheets indicates that either the patients did not know what an ADR was or did not feel inclined to self-report as they felt that the researcher may judge them or due to stigmatization of a condition e.g. depression was under reported by the patients. There were significantly higher recorded cases of depression than those that were self-reported (p<0.05). This finding is similar to a study conducted by Sirey *et al*. which illustrated the stigma associated with depression [38]. On the other hand, the patient may have thought it to be an expected side effect associated with medicine, and did not report it to the doctor. Under reporting of ADRs still remains a global problem [39]. Another commonly reported side effect as though it was an ADR was fever. It is therefore essential for HCPs to educate and inform patients on the difference between ADRs and side effects, so that there is consistency in terminology and there is proper reporting and management of ADRs. The discrepancies were picked up in this study because of the 2 methods of data collection. Therefore, it is important when self-reported studies of this nature are conducted, they should be backed up by retrospective or qualitative studies. The importance of proper data collection with regard to patient ADR management is vital, and also for strengthening of pharmacovigilance activities.

Limitations: even though the study provided an understanding of the number of ADRs experienced by HIV infected patients in three public sector facilities, it did however have a number of limitations. The loss of patient files was encountered and posed a barrier in recording ADRs experienced by patients, hence resulting in exclusion of the participant patient. The referral system, where patients are seen in a hospital once every 6 months, also gave inadequate data in some instances. During the development of the study tool, the psychometric testing was not carried out and that was one of the limitations in the study.

## Conclusion

Adverse drug reactions were experienced by the majority of surveyed patients. All regimens initiated were implicated in causing the ADR with the first line regimen (Tenofovir (TDF), Emtricitabine (FTC) and Efavirenz (EFV) initiated in 2012) having the most number of patients experiencing an ADR. Much education on what an ADR is versus a side effect needs to be effected so that patients identify and report an ADR correctly. This will further strengthen pharmacovigilance activities leading to effective management of ADR implicated morbidity and mortality.

### What is known about this topic

- Cross-sectional studies have shown that ADRs associated with HAART are prevalent in HIV patients.

### What this study adds

- Through the use of retrospective analysis and cross-sectional analysis methods, our study validates ADRs reported by patients by contrasting with those recorded by HCPs in medical files; the findings in our study show that in some cases, patients report expected side effects as ADRs therefore patients ought to be educated on ADRs.

## References

[1] Joint United Nations Programme on HIV/AID (2010). Global report: UNAIDS report on the global AIDS epidemic 2010.

[2] Avert (2020). Global information and education on HIV and AIDS. HIV and AIDS in South Africa.

[3] Vagiri R, Meyer JG, Gustav B, Stephanus AG (2018). Research relationship between adherence and health-related quality of life among HIV-patients in South Africa: findings and implications. Journal of AIDS and HIV Research.

[4] Luma HN, Doualla M, Choukem S, Temfack E, Ashuntantang G, Joko HA (2012). Adverse drug reactions of highly active antiretroviral therapy (HAART) in HIV infected patients at the General Hospital, Douala, Cameroon: a cross sectional study. Pan African Medical Journal.

[5] Mehta U, Kalk E, Boulle A, Nkambule P, Gouws J, Rees H (2017). Pharmacovigilance: a public health priority for South Africa. S Afr Health Rev.

[6] Edwards R, Aronson J (2000). Adverse drug reactions: definitions, diagnosis and management. Lancet.

[7] Nebeker J, Barach P, Samore M (2004). Clarifying adverse drug events: a clinician's guide to terminology, documentation, and reporting. Ann Intern Med.

[8] VA Center for Medication Safety And VHA Pharmacy Benefits Management Strategic Healthcare Group and the Medical Advisory Panel (2006). Adverse drug events, adverse drug reactions and medication errors: frequently a asked questions.

[9] South African Health Products Regulatory Authority Reporting Guidelines (2020). Guideline for adverse drug reactions (ADRs) reporting for healthcare professsionals. Communication to Healthcare Professionals.

[10] Lagnaoui R, Moore N, Fach J, Longy-Boursier M, Bégaud B (2000). Adverse drug reactions in a department of systemic diseases-oriented internal medicine: prevalence, incidence, direct costs and avoidability. Eur J Clin Pharmacol.

[11] Pirmohamed M, James S, Meakin S, Green C, Scott AK, Walley TJ (2004). Adverse drug reactions as cause of admission to hospital: prospective analysis of 18,820 patients. BMJ.

[12] Masenyetse L, Manda S, Mwambi H (2015). An assessment of adverse drug reactions among HIV positive patients receiving antiretroviral treatment in South Africa. AIDS Res Ther.

[13] Srikanth A, Babu C, Yadav HN, Jain SK (2012). Incidence of adverse drug reactions in human immune deficiency virus-positive patients using highly active antiretroviral therapy. J Adv Pharm Technol Res.

[14] Mouton J, Smidt MF, Jobanputra N, Mehta U, Stewart A, de Waal R (2020). Serious adverse drug reactions at two children's hospitals in South Africa. BMC Pediatr.

[15] Kindie E, Anteneh ZA, Worku E (2017). Time to development of adverse drug and associated factors among adult HIV positive patients on antiretroviral treatment in Bahir Dar city, North West Ethopia. PLoS One.

[16] Van Graan R, Viljoen M, Rheeders M, Motara F (2018). Retrospective clinical analysis of adverse drug reactions associated with antiretroviral therapy in Tlokwe district, South Africa. South African Family Practice.

[17] Mehta U, Durrheim D, Blockman M, Kredo T, Gounden R, Barnes KI (2008). Adverse drug reactions in adult medical inpatients in a South African hospital serving a community with a high HIV/AIDS prevalence: prospective observational study. Br J Clin Pharmacol.

[18] Domingo P, Lozanode F (2011). Management of antiretroviral drug toxicity. Enferm Infecc Microbiol Clin.

[19] Li H, Marley G, Ma W, Wei C, Lackey M, Ma Q (2017). The role of ARV associated adverse drug reactions in influencing adherence among HIV infected individuals: a systematic review and qualitative meta synthesis. AIDS Behav.

[20] Mberi NM, Kuonza LR, Dube NM, Nattey C, Manda S, Summers R (2015). Determinants of loss to follow-up in patients on antiretroviral treatment, South Africa, 2004-2012: a cohort study. BMC Health Serv Res.

[21] Oumar AA, Abdoulaye A, Maiga M, Sidibé Y, Cissoko Y, Konaté I (2017). Adverse drug reactions to antiretroviral therapy (ART): study in HIV infected Adults in SIkasso (Mali). J Pharmacovigil.

[22] Manickum VK, Suleman F (2012). Evaluating adverse drug reactions among HAART patients in a resource-constrained province of South Africa. Afr J AIDS Res.

[23] Ethekwini municipality Ethekwini district AIDS council quarter 1, 2017/2018 report.

[24] Cochran WG (2007). Sampling techniques. John Wiley & Sons.

[25] (2011). Statistics South Africa. Census in brief Census.

[26] Tadesse WT, Mekonnen AB, Tesfaye WH, Tadesse YT (2014). Self-reported adverse drug reactions and their influence on highly active antiretroviral therapy in HIV infected patients: a cross sectional study. BMC Pharmacol Toxicol.

[27] Mendes JC, Bonolo PF, das Graças M, Ceccato B, Costa JO, Reis AMM (2018). Adverse reactions associated with first-line regimens in patient initiating antiretroviral therapy. Eur J Clin Pharmacol.

[28] Zucker I, Prendergast B (2020). Sex differences in pharmacokinetics predict adverse drug reactions in women. Biol Sex Differ.

[29] Oscanoa T, Lizaraso F, Carvajal A (2017). Hospital admissions due to adverse drug reactions in the elderly. A meta-analysis. Eur J Clin Pharmacol.

[30] Jain A, Lihite R, Lahkar M, Baruah S (2016). A study on adverse drug reactions to first-line antiretroviral therapy in HIV infected patients at a tertiary care hospital in Northeast India. HIV & AIDS Review.

[31] Hagos L, Fessehaye S, Anand IS (2019). Nature and prevalence of adverse drug reactions of antiretroviral medications in Halibet National Referral hospital: a retrospective study. BMC Pharmacol Toxicol.

[32] Muñoz-Moreno J, Fumaz C, Ferrer M, González-García M, Moltó J, Negredo E, Clotet B (2009). Neuropsychiatric symptoms associated with efavirenz: prevalence, correlates, and management. A neurobehavioral review. AIDS Rev.

[33] Shubber Z, Calmy A, Andrieux-Meyer I, Vitoria M, Renaud-Théry F, Shaffer N (2013). Adverse events associated with nevirapine and efavirenz-based first-line antiretroviral therapy: a systematic review and meta-analysis. AIDS.

[34] Decloedt E, Maartens G (2013). Neuronal toxicity of efavirenz: a systematic review. Expert Opin Drug Saf.

[35] Islam F, Wu J, Jansson J, Wilson D (2012). Relative risk of cardiovascular disease among people living with HIV: a systematic review and meta-analysis. HIV Med.

[36] Areechokchai D, Bowonwatanuwong C, Phonrat B, Pitisuttithum P, Maek-A-Nantawat W (2009). Pregnancy outcomes among HIV-infected women undergoing antiretroviral therapy. Open AIDS J.

[37] Anastasi JK, Bernadette C (2011). Nausea and vomiting in HIV/AIDS. Gastroenterol Nurs.

[38] Sirey JA, Bruce ML, Alexopoulos GS, Perlick DA, Friedman SJ, Meyers BS (2001). Stigma as a barrier to recovery: perceived stigma and patient-rated severity of illness as predictors of antidepressant drug adherence. Psychiatr Serv.

[39] Bogolubova S, Padayachee N, Schellack N (2018). Knowledge, attitudes and practices of nurses and pharmacists towards adverse drug reaction reporting in the South African private hospital sector. Health SA.

